# Optic disc granuloma with epididymitis: a diagnostic challenge

**DOI:** 10.1186/s12348-017-0131-6

**Published:** 2017-05-22

**Authors:** Parthopratim Dutta Majumder, Sowkath Ali, Jyotirmay Biswas

**Affiliations:** Medical and Vision Research Foundation, Sankara Nethralaya, 18, College Road, Chennai, 600 006 India

**Keywords:** Tuberculosis, Sarcoidosis, Epididymitis, Azoospermia, Optic disc granuloma

## Abstract

**Background:**

Sarcoidosis and tuberculosis (TB) share epidemiological, immunological, and molecular features common to both, and often, it becomes difficult to differentiate them especially in a highly TB-endemic country like India. We report a case of optic disc granuloma along with epididymitis and infertility in a male who showed a response to treatment with anti-tuberculosis therapy and steroids.

**Results:**

A 35-year-old Indian male who presented with complaints of blurring vision in the right eye for 6 months and treated elsewhere with two intravitreal injections of anti-VEGF was found to have optic disc granuloma in the right eye. His investigations revealed highly positive Mantoux test, negative QuantiFERON test, elevated serum ACE, and HRCT of chest showing features of sarcoidosis. He also gave a history of primary infertility. Ultrasound Doppler of the scrotum was suggestive of epididymitis. After getting a chest physician opinion, the patient was started on the first line of ATT followed by oral steroids. On subsequent follow-up visits, the patient showed resolution of lesions along with the return of fertility.

**Conclusions:**

The index case of optic disc granuloma along with epididymitis had features of both tuberculosis and sarcoidosis and falls in the gray zone of tuberculous sarcoidosis and showed a response to both ATT and corticosteroids. We report this case report for its unique presentation.

## Introduction

Sarcoidosis and tuberculosis share epidemiological, immunological, and molecular features common to both, and often, it becomes difficult to differentiate them especially in a highly endemic country like India [1, 2]. Ocular involvement in sarcoidosis has a plethora of presentations and varies widely from 13 to 79% [1, 3]. Optic nerve head involvement in sarcoidosis is rare [4], and even rarer is the genitourinary involvement in sarcoidosis, accounting for less 0.2% of the cases [5]. On the other hand, optic nerve head granuloma in tuberculosis is rare, but not uncommon. [6] Tuberculosis is a well-documented cause of male infertility in developing countries [7]. We report a case of optic nerve head granuloma presenting with epididymitis and infertility in a male who had been on oral corticosteroid and anti-tuberculosis treatment and showed a dramatic response to therapy.

## Case report

A 35-year-old male presented to our clinic with a 6-month history of blurred vision in his right eye. He was diagnosed to have vascular lesion over the optic disc elsewhere and was treated with intravitreal injection of bevacizumab twice and a course of oral steroid (40 mg/day), but symptoms had not improved. The patient also gave a 2-year history of primary infertility for which he and his spouse consulted a doctor recently, and his semen analysis revealed azoospermia. A detailed past medical history was taken and revealed that the patient had been exposed to TB in his early childhood when his uncle died due to tuberculosis of the bone.

**Fig. 1:**
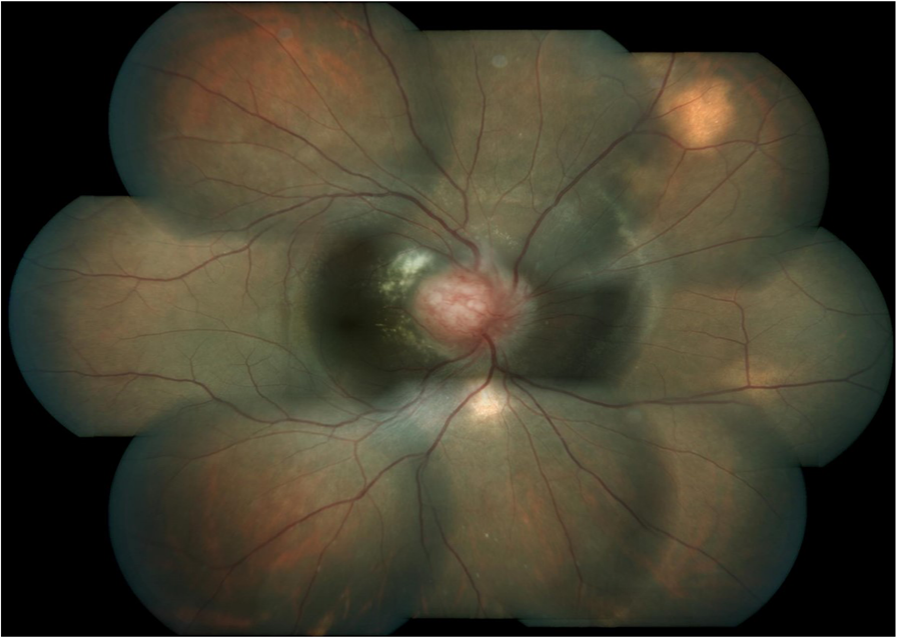
Fundus picture of the right eye showing vascularized elevated granuloma involving the optic disc with minimal peripapillary subretinal fluid and a choroidal nodule in the superonasal quadrant of the eye

**Fig. 2:**
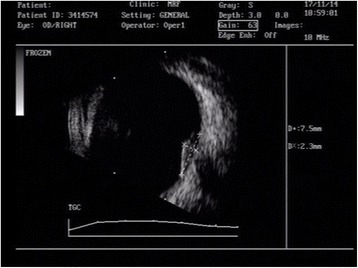
Ultrasound B-scan of the right eye

**Fig. 3:**
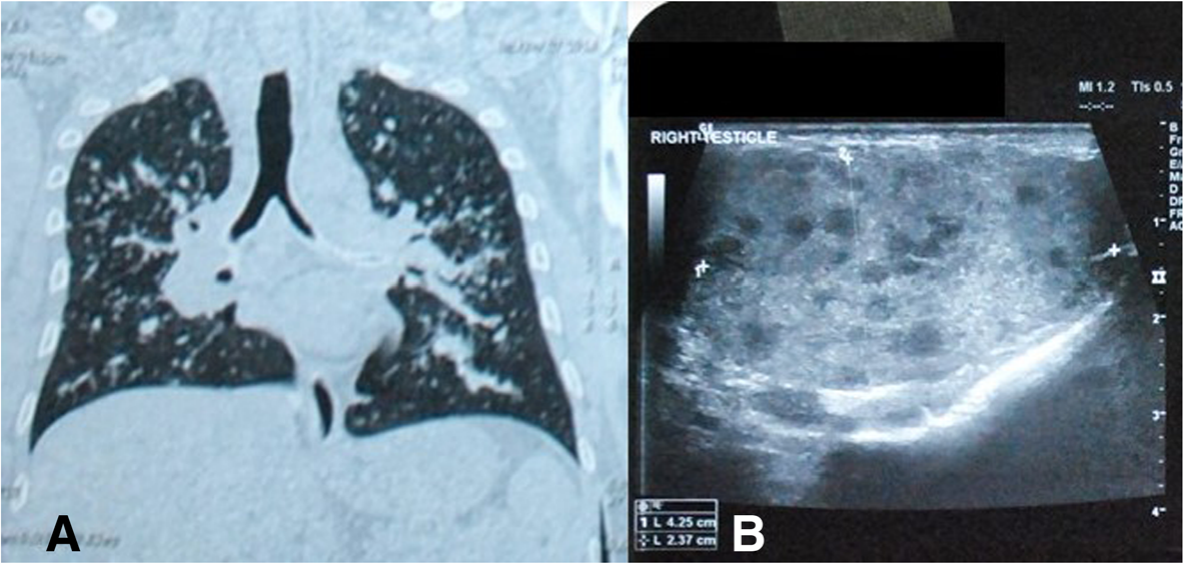
**a.** HRCT chest showing multiple areas of nodular consolidation and nodules along the fissures, mediastinal and hilar lymphadenopathy, and bilateral basal pleural thickening **b.** Ultrasound Doppler study of scrotum showing mild epididymis echotexture with small cysts

On examination, his best-corrected visual acuity was 6/12 and 6/6 in the right and left eye, respectively, (Snellen). Slit lamp examination revealed quiet anterior chamber and cells in anterior vitreous in both eyes. Fundus examination of the right eye showed vascularized elevated granuloma involving the optic disc with minimal peripapillary subretinal fluid and a choroidal nodule in the superonasal quadrant of the right eye (Fig. 1). Examination of the left eye fundus was unremarkable. Ultrasound B-scan of the right eye documented localized area of retino-choroidal elevation over the optic nerve head (8.2 × 2.3 × 7.5 mm) with no evidence of calcification or choroidal excavation (Fig. 2). HRCT chest showed multiple areas of nodular consolidation and nodules along the fissures, mediastinal and hilar lymphadenopathy, and bilateral basal pleural thickening—all pointing towards sarcoidosis (Fig. 3).

His Mantoux test (5 tuberculin units, 0.1 ml) was 20 × 20 mm and interferon gamma release assay (QuantiFERON-TB Gold) was negative. Serum ACE was elevated, and serum lysozyme was within normal limits. Ultrasound Doppler study of the scrotum showed mild epididymis echotexture with small cysts suggestive of chronic epididymitis (Fig. 3b). The patient refused to undergo a testicular biopsy and insisted on medical management. He was sent to a chest physician, who had asked for fiberoptic bronchoscopy to confirm the diagnosis, which the patient declined again. The patient was started on first line ATT by the chest physician followed by oral steroids 60 mg/day in tapering doses.

**Fig. 4:**
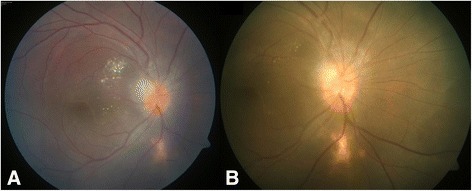
**a** & **b**: fundus photograph of the right eye showing resolved lesions at 3-month and 8-month of follow-up visit

The patient responded very well with his BCVA improving to 6/7.5 and 6/6 in the right and left eye, respectively, with fundus showing resolved lesions with ILM folds in the right eye. *S*wept-source optical coherence tomography (SS-OCT) showed resolution of the lesion with a thick membrane over the disc, epiretinal membrane over the macula with a normal foveal thickness in the right eye (Fig. 4a). The patient has seen again after 8 months (Fig. 4b). Interestingly, the patient was found to be oligospermic at 6 months of follow-up and informed us about his wife’s conception.

## Discussion

We present a case with the rare finding of optic nerve head granuloma and the even rarer finding of infertility related to epididymitis due to chronic granulomatous etiology. In our patient, raised serum ACE and findings in HRCT chest was suggestive of possible sarcoidosis whereas highly positive Mantoux test, positive history of contact with tuberculosis patient, and response to ATT were in favor of tuberculosis. The lack of tissue biopsy in our case made the definitive diagnosis difficult which was further compounded by the laboratory investigations.

Rohrberg et al [8] described that a negative TST, in sarcoidosis patients, as is true for general patients, should never rule out tuberculosis. However, a positive test in a sarcoid patient, given its high specificity and the seriousness of tuberculosis, should prompt additional work-up for tuberculosis. Also, positive tuberculin test does not rule out the diagnosis of sarcoidosis [9]. In areas where tuberculosis is endemic, it has been suggested that ELISA-IGRA offers increased specificity in the diagnosis of *Mycobacterium tuberculosis* infection at the cost of some sensitivity [10]. In fact, in head-to-head comparisons between the TST and interferon gamma release assay, the highest sensitivity corresponded to the TST, followed by interferon gamma release assay based on PPD, and lastly interferon gamma release assay based on specific region of difference 1 antigens (such as ELISA-IGRA) [10]. The drawback of IGRA being it cannot differentiate between active and latent tuberculosis. Chakrabarti et al [11] found that combined use of HRCT chest and serum ACE levels can diagnose ocular sarcoidosis in 95% of patients and may be useful in patients who are not comfortable to undergo biopsy. Serum ACE is a nonspecific marker but in our case, we relied upon HRCT and took ACE as a supportive tool to diagnosis sarcoid. Also, ONH involvement is relatively uncommon, though not rare, in ocular tuberculosis. Genitourinary involvement by sarcoidosis is very rare, accounting for 0.2% of cases. However, infertility due to epididymal sarcoidosis is usually reversible and shows good result following anti-inflammatory therapy [12, 13]. On the other hand, genitourinary tuberculosis is relatively more common and accounts for 20–73% of all cases of extra-pulmonary tuberculosis in the general population [14]. Tuberculous epididymo-orchitis accounts for 22% of all cases of genitourinary tuberculosis [14]. Baring few case reports [15], the treatment of infertility associated with genitourinary tuberculosis is usually frustrating and in most of the cases, fertility does not return to normal following anti-tuberculosis treatment [16, 17]. Our patient was thoroughly examined by a chest physician, who also felt the need of ATT in a patient with exposure to tuberculosis patient and where a laboratory result reflects both sarcoidosis and tuberculosis.

There is a considerable overlap between clinical presentations of sarcoidosis and tuberculosis, and there exists a gray zone wherein we find patients that fulfill diagnostic criteria of both tuberculosis and sarcoidosis. Scadding was the first in 1962 to introduce the term “tuberculous sarcoidosis” for this category of patients [14]. Various authors have postulated that tuberculosis and sarcoidosis are a spectrum of the same disease, the difference in the immunological response of the host leading to different manifestations [15]. Agarwal et al [1] further proposed a subdivision classification for both sarcoidosis and tuberculosis—sarcoidosis, sarcoid-tuberculous, tuberculous-sarcoid, and tuberculosis based on molecular, immunological, and histopathological aspects. They also proposed that the sarcoid-tuberculous and tuberculous-sarcoid groups should be treated with both immunosuppressive agents and anti-tuberculosis medications to avoid re-activation or undertreatment of the other.

We believe that our case represent a gray zone of both tuberculosis and sarcoidosis-possibly tuberculous sarcoidosis and highlights the effectiveness of combined immunosuppressive and anti-tuberculosis therapy in such case scenario. Gupta et al. [18] reported a 23-year-old man who presented with choroidal tuberculoma and epididymitis and PCR-analysis of the vitreous, and epididymal fluid using primers specific for the IS6110 sequence of *M. tuberculosis* was positive. The sequence analysis of both PCR products yielded the same sequence, indicating the fact that the same pathogen was responsible for the epididymal and choroidal infections. In another case report, Dutta et al. [19] reported a 38-year-old male who presented with bilateral testicular swelling, night sweats, and weight loss and was initially diagnosed with tuberculosis and treated with anti-tubercular therapy. His testicular biopsy sample was suggestive of necrotizing granulomatous inflammation with no microbiological evidence of mycobacteria. Lack of response to anti-tubercular therapy and clinical deterioration led to further investigations, which came out positive for sarcoidosis. The authors emphasized the importance of considering sarcoidosis even when biopsies show granulomata with patchy necrosis that mimics tuberculosis. The main limitation of our case report is that we could not perform a histopathogical examination of bronchial aspirate or testicular biopsy sample, as our patient declined to undergo any of them.
